# A cautionary tale for health education initiatives in vulnerable populations: Improving nutrition in Haiti prisons

**DOI:** 10.3389/fmed.2022.1076583

**Published:** 2022-12-20

**Authors:** Arch G. Mainous, Jean Bernard, Stephanie Auguste, Jacques R. Louis, Danove J. Dieufort, Karine Duverger, Madsen Beau de Rochars, John May

**Affiliations:** ^1^Department of Health Services Research, Management, and Policy, University of Florida, Gainesville, FL, United States; ^2^Department of Community Health and Family Medicine, University of Florida, Gainesville, FL, United States; ^3^Health through Walls, Port-Au-Prince, Haiti; ^4^School of Medicine and Pharmacy, Université d'État d'Haïti (UEH), Port-Au-Prince, Haiti; ^5^Centurion Health, Sterling, VA, United States

**Keywords:** Haiti, nutrition, health education, low-income and middle-income countries, social strife

## Abstract

**Introduction:**

Prisons in low-income countries have barriers to providing adequate nutrition to the incarcerated. This perspective discusses a quality improvement program with health education to improve nutrition provided to men in two prisons in Haiti.

**Methods:**

Incarcerated men in the National Penitentiary in Port Au Prince and the prison in Mirebalais were the focus of the program. A culturally competent educational intervention was delivered to the prison cooks. Program evaluation included a baseline and a follow-up assessment in 2021 and 2022 in both prisons. Calories, body composition, and nutrition were assessed at both time points.

**Results:**

Among 1,060 men assessed in the baseline time period, the mean number of calories per day was 571. Further, 62.5% had a vitamin C intake insufficient to prevent scurvy and 91.6% had vitamin B1 insufficient to prevent beriberi. In the follow-up period, caloric intake decreased to a mean of 454 per day (*p* < 0.001). The proportion of incarcerated men who had insufficient vitamin C and vitamin B1 to prevent disease increased in the follow-up period.

**Discussion:**

The caloric and nutritional intake of incarcerated men in Haitian prisons is poor and is getting worse. The educational intervention with the cooks was not successful due to civil and political strife in the low-income country of Haiti. Standard interventions to improve nutrition need to consider the social context for accessing food.

## Introduction

Food insecurity is an issue that affects people's health throughout the world (1, 2). In addition to having enough food to eat to alleviate hunger, people need access to food that will promote health and prevent disease. Specifically, there is an expectation of nutritional adequacy as well as simply caloric sufficiency. Low-income countries are particularly affected by a lack of nutritious food (2). One estimate suggests that about half of the people in low-income countries experience food insecurity compared to only 10% of the population in high-income countries (3).

Persons who are detained or imprisoned by authorities of the justice system depend on food being provided by the institution and are vulnerable to food and nutrition insecurity. They are unable to freely choose their food or access different foods. Consequently, the prison and the prison cooks are important gatekeepers in selecting and preparing food.

A review of studies on currently incarcerated persons showed mixed results with some showing that currently incarcerated persons may actually exceed recommended levels of calories in higher-income countries (4). However, the total caloric intake for prisoners in low-income and middle-income countries was below the recommended daily allowance (RDA). One study on incarcerated prisoners in the Ivory Coast showed not only were there insufficient calories in the diet but there was inadequate nutrition, as evidenced by an outbreak of beriberi, a result of inadequate vitamin B1 (5). Similarly, an investigation in a prison in Ethiopia showed that the diet provided to the prisoners excluded fruits and vegetables which was directly related to a high prevalence of scurvy (6).

Health initiatives with vulnerable populations many times focus on health education (7, 8). The underlying assumption is that with better education on a healthy diet that individuals will have better nutrition consistent with disease prevention. In the case of prisons, it would follow that the people to be educated would be those choosing and preparing the food.

Haiti is a low-income country that is the poorest in the Western Hemisphere. As such, incarcerated persons in Haiti may have an increased vulnerability to food and nutrition insecurity. We implemented a quality improvement program in two Haitian prisons designed to improve the nutrition received by the incarcerated men. The program was focused on nutrition education for the prison cooks and the prison administration.

## Methods

Our quality improvement, health education initiative took place for adult men in two (2) of Haiti's 17 prisons, the Penitencier National and the Mirebalais prison. Penitencier National is the largest prison in Haiti, detaining more than 4,000 adult men in a space intended for 800. The Mirebalais prison holds more than 300 persons but was designed for a smaller population. We conducted a baseline assessment in 2021, implemented a quality improvement program with education for the prison cooks, and then performed a follow-up assessment in 2022. This program evaluation was approved by the University of Florida Institutional Review Board as quality improvement.

### Nutritional assessment

Prior to planning and implementing nutritional interventions in the prisons, it is critical to understand the diet of those people presently incarcerated. This includes both the food provided by the prison and food provided by the family or other sources of food (e.g., markets set up by individuals who are also living in prison) to individuals living in prison. A 24-h dietary recall was used to assess nutrition because it is the most widely used strategy for dietary intake data (9). This structured interview is intended to capture detailed information about all foods and beverages consumed by the respondent in the past 24 h. Computer programs have been successfully used in nutritional assessments of people living in prison (10). We used the Automated Self-Administered 24-H (ASA24^®^) Dietary Assessment Tool which was designed by the National Cancer Institute.

The food provided by the prison was assessed through the categorization of the recipes used in food preparation and delivery to those living in the prison. This included assessments based on serving size. In addition to calories, the assessment provided indications of the proportion of nutritional components such as carbohydrates, fats, protein, sodium, calcium, and vitamins.

Although the Recommended Dietary Allowance (RDA) for vitamin C is 75 mg for women and 90 mg for men the amount of Vitamin C needed to prevent scurvy is 10 mg/day (11). The amount of Vitamin B1 needed to prevent beriberi is 1.2 mg/day (12).

### Anthropometric examination

Body mass index, a person's weight in kilograms divided by the square of height in meters, is the most commonly used strategy for determining malnutrition. These evaluations were collected in an exam and were not provided by self-report of the incarcerated. Individuals with a BMI <18.5 are considered to be underweight and potentially malnourished. BMI and age were assessed in the exam.

Two medical students from the State University medical school in Port-Au-Prince were engaged as research associates on the project. They were fluent in French and Haitian Kreyol and local food. This allowed them to effectively discuss dietary history with the prisoners. Further, they were trained on how to use the Automated Self-Administered 24-h (ASA24^®^) Dietary Assessment Tool from the National Cancer Institute for the collection of nutrition data. We trained them on the general procedures and how to use the tablet computers for data entry and how to collect height and weight.

### Nutrition education intervention

Following the baseline assessments, we focused on nutritional deficits to devise an educational intervention to improve nutrition in prisons. We gathered information in structured interviews from the cooks at the National Penitentiary and the prison in Mirebalais about their cooking activities. This included specifics on recipes for common dishes created for the incarcerated like Riz National. Other recipes included Ble with beans, Bouillie, and Corn meal. We gathered specific data on quantities of ingredients in the recipes, techniques for cooking as well as how many individuals each batch was designed to feed. We also collected a variety of other information including questions about the preparation of perishable foods that might provide more and varied nutritional content like vegetables and meat. It was clear that without a freezer meat preparation was not reliable.

We analyzed the information provided by the cooks and used it to create an intervention for the cooks to try and improve the nutritional content of the food served. We gathered additional feedback from the cooks on the proposed intervention regarding the feasibility of the suggested cooking changes. This included a discussion of the ability to access different foods, the logistics of cooking different things, and the potential acceptance of the new food by incarcerated persons.

Some of the constraints in Haiti that plague the society affected our program early on. The significant gang violence and the lack of fuel affected our ability to do the training with the prison cooks. We conducted training for the medical students from UEH as well as personnel from Health through Walls, a non-governmental organization supporting prison healthcare systems. The training was undertaken with these individuals and they would then train the prison cooks.

The training was based on nutritional deficits identified in the initial baseline assessment of persons in the National Penitentiary in Port-Au-Prince and the prison in Mirebalais. The training was designed to improve knowledge of nutrition and in particular, discuss foods that would address the nutritional deficits of vitamin B1 deficiency and affiliated beriberi disease and vitamin C deficiency and affiliated scurvy.

Realizing that some foodstuffs were limited (e.g., fresh vegetables and meat) we recommended changes to the types of rice or beans to increase the nutritional content. This educational intervention was delivered in November 2021 to the cooks at both prisons. We were aware of the financial constraints on the prison system for buying food so we focused a lot on recipes with reasonable food substitutes (e.g., brown rice or enriched rice rather than white rice) rather than just educating the personnel about adding more food. Adding such food would help to address both the caloric deficits but also the nutritional deficits. We talked about foods that may be available to combine into current culturally acceptable recipes that were rich in vitamin C, vitamin B1, lycopene, vitamin K, vitamin B6, and vitamin B12. The National Penitentiary is scheduled to receive freezers and refrigerators so we also discussed the potential of adding ingredients like yogurt to stews or sauces because of the high protein content and inclusion of calcium and vitamin D.

### Follow-up assessment and evaluation of the success of the health education program

We conducted follow-up assessments in March through June 2022 using the same strategy and methods as the initial baseline assessments. Descriptive statistics of the nutrition data for the incarcerated were created to summarize differences between the pre-intervention and post-intervention data in 2021 and 2022. Comparisons between the two time points were made using the *t*-tests for continuous variables and Chi-square tests for categorical variables. All analyses were conducted using SAS 9.4.

## Results

In the baseline assessment, 560 men were interviewed and examined from the two prisons (400-Penitencier; 160—Mirebalais) while 500 men (400—Penitencier; 100—Mirebalais) were interviewed in the follow-up assessment. Table 1 shows the results from both the baseline and follow-up assessments. The mean caloric intake per day in both the baseline and the follow-up was <600 calories, a level consistent with a starvation-level diet. Importantly, the number of calories consumed per day showed a statistically significant decrease between the baseline and the follow-up.

**Table 1:** Comparison of baseline and follow-up nutritional assessments combined for both prisons.

	**2021**	**2022**	***p*-values**
Total	560	500	
**Age**
18–29	221 (39.5%)	215 (43.0%)	
30–59	319 (57.0%)	255 (51.0%)	
60+	20 (3.5%)	30 (6.0%)	*p* > 0.05
**BMI**
Underweight ( ≤ 18.5)	358 (63.9%)	181 (36.2%)	
Normal weight (18.6–24.9)	199 (35.5%)	267 (53.4%)	
Overweight (25–29.9)	3 (0.5%)	47 (9.4%)	
Obese (30+)	0 (0.0%)	5 (1.0%)	*P* < 0.001
Number of meals per day (mean ± SD)	1.63 ± 0.55	1.16 ± 0.43	*P* < 0.001
Daily caloric intake (mean ± SD)	571 ± 210	454 ± 206	*P* < 0.001
Men who receive 1 meal per day	223 (39.8%)	429 (85.8%)	*P* < 0.001
Men whose daily vitamin C intake is insufficient to prevent scurvy	350 (62.5%)	467 (93.4%)	*P* < 0.001
Men whose daily thiamine intake is insufficient to prevent symptoms of beriberi*	513 (91.6%)	494 (98.8%)	*P* < 0.001

In terms of nutritional security, the intake of vitamin C (scurvy) and vitamin B1 (beriberi), a majority of the incarcerated men had an inadequate daily intake of both vitamin C and vitamin B1. As with caloric intake, the nutritional composition of the diet actually was statistically significantly worse in the follow-up assessment compared to the baseline.

## Discussion

What did we learn from our health education initiative? The caloric intake and nutritional composition of the diet of persons incarcerated in Haiti are poor. It is possible that the high prevalence of underweight incarcerated men was due to underlying medical conditions but that seems unlikely based on the measured diet provided to the men. The baseline nutritional assessment of persons within the Haitian prisons indicated that they have a starvation-level diet, are underweight, and have significant nutritional deficiencies. Particularly concerning is that during the time period of our health education program, the nutritional composition of the diet in the prisons substantially worsened. The average number of calories per day for persons in both prisons showed a statistically significant decrease. The average number of meals persons received each day substantially decreased as well. These results suggest an extremely high risk of poor health outcomes for those living within the prisons.

As well-meaning as our program was to help a vulnerable population in a low-income country with social strife it became clear that our educational training of the prison cooks was not the key to improving the nutrition and health of the incarcerated men. During the time of the project, political unrest increased in Haiti. Haiti's President, Jovenel Moise, was assassinated on 7 July 2021 and the position of President remains unfilled. Violence and chaos have overtaken many communities, and family members who previously visited the prison and provided nutritional assistance are unable to travel (13). Although violence has until recently been most pronounced in Port au Prince, the location of the Penitencier National, other areas of the country have also been affected. Mirebalais, the location of the other investigated prison has also experienced violence, and driving from Port au Prince to Mirebalais can be dangerous. This affects the delivery of food to the prisons. In a country with significant criminal gang activity and a low tax base for the provision of social services, the selection of food and delivery of food to prisons is challenging.

In addition to a starvation-level diet, which got statistically significantly worse during the examination period, the nutritional components of the diet also got statistically significantly worse during the study period. The number of prisoners at risk of scurvy or beriberi substantially increased. In the follow-up assessment, more than 75% of the men had a daily diet that put them at risk of both scurvy (lack of Vitamin C) and beriberi (lack of Vitamin B1). These observations suggest that persons incarcerated at both the Penitencier National and Mirebalais were not receiving enough food to support a healthy diet and what they were receiving was not appropriate nutrition. Our validated computer program that assessed the 24-h dietary history of the incarcerated men was able to record and evaluate the nutritional content of thousands of foods but our data showed that the diet and nutrition in the prisons were very narrow and limited. Fruits and vegetables beyond dried beans were essentially non-existent in the diet. Many nutritional deficiencies like scurvy and beriberi previously documented in Haitian prisons have worsened, while quite rare in the general population in high-income countries because of the distribution of nutritious food like fruits and vegetables (14, 15).

The educational intervention with the cooks in the two participating prisons was meant to improve the composition of the diets of those in prison. The intervention was unsuccessful for a variety of reasons. First, it became clear that educating the cooks and providing recipes for a more healthy diet was based on the assumption that the cooks had some ability to plan meals and determine the ingredients in the dishes they prepared. In reality, the cooks were greatly dependent upon what was delivered to the prison and had to cook what was delivered. The prisons are quite crowded. Clearly, this is a problem if food delivery is insufficient to provide even a starvation-level diet. Second, the overall caloric intake was so low that substitution recipes (e.g., use a different type of rice or beans) even if they were implemented were likely to have only a marginal impact on vitamins and nutrition. The intervention was designed so that substitutions were culturally consistent and they were foods that should be available in Haiti. Again, if the amount of food is so low and the cooks cannot choose what they prepare then it is disappointing but not surprising that the nutritional composition of the diet did not improve. Third, the style of cooking in large pots required a greater diversity of foodstuffs within the stews than what was available to yield a nutritional impact across the numerous persons to feed and the small serving size. Cooking in large pots is consistent with a strategy to feed a large number of people at each meal. However, the narrow list of ingredients limited nutrition. Fourth, there was no consistent serving size for the persons. Because of security issues, each person brings their own plastic bucket and those vary in size. There are likely sanitation and corresponding health issues that are not being addressed in the creation and distribution of food in this way.

It should be noted that it was difficult to conduct the follow-up nutritional assessment in the Penitencier National and Mirebalais due to violence in the society. The Penitencier National was locked down many times and the research staff were not allowed in to interview and weigh and measure the incarcerated persons. On several occasions, those incarcerated would go 2–3 days without food during these lockdowns. It was also difficult to get to the Mirebalais prison due to criminal gang violence on the road to Mirebalais. Because our validated nutritional assessment computer program, which is endorsed and distributed by the National Cancer Institute, is based on a 24-h dietary history, our results, however dramatic are likely an overestimate of caloric intake. So our results are potentially skewed showing more calories than is likely for an average day because we were only allowed to collect data and include days in which they ate.

## Future Directions

An initiative that may have a particular utility that should be considered is to create a farming system at or near the prison where inmates could grow their own food. This would provide the opportunity to produce food for a more varied and nutritious diet. It would also eliminate some of the challenges dealing with food vendors and their ability to supply and transport food in a dangerous environment. A steady source of food would be ensured for the prison. It would also decrease the impact of potential budget cuts on the food supply to the prison. In fact, a pilot program to try and institute such a system has been conceptualized and is in the early stages at the prison at Fort Liberté on the northern coast of Haiti. This system would work for prisons in rural areas. Translating this model to the Penitencier National in the center of Port au Prince will require a creative solution merging the self-sufficiency provided by a prison farm with the space constraints of an urban area.

In conclusion, identification of the factors preventing Haiti's prisons from obtaining enough food and the ability for cooks to plan nutritious meals will have the greatest effect on the health and wellbeing of those persons within Haitian prisons. Issues in the provision of food and specifically the lack of food are particularly disturbing and require immediate solutions. During the project, the caloric and nutritional composition of the diet was low and got worse. It is important that the cooks have sufficient food for those who are incarcerated and have some ability to choose what they cook. Violence and crime in Hatian society and the corresponding context of obtaining food, transporting it, and paying for it in a situation of violence and social chaos requires planners to think creatively and consider how to deal with these significant challenges. If not, educational interventions are unlikely to be successful.

## Data availability statement

The original contributions presented in the study are included in the article/supplementary material, further inquiries can be directed to the corresponding author.

## Author contributions

AM wrote the first draft. All authors contributed to the project and reviewed the manuscript drafts.
